# Variable-Temperature ^1^H-NMR Studies on Two *C*-Glycosylflavones

**DOI:** 10.3390/molecules17077914

**Published:** 2012-07-02

**Authors:** Julia H. Frank, Yomica L. Powder-George, Russel S. Ramsewak, William F. Reynolds

**Affiliations:** 1 Department of Chemistry, The University of the West Indies, St. Augustine, Trinidad and Tobago, West Indies; Email: modelle_cc@hotmail.com (J.H.F.); pyomica@hotmail.com (Y.L.P.-G.); 2 Department of Chemistry, University of Toronto, Toronto, ON M5S 3H6, Canada

**Keywords:** variable-temperature ^1^H-NMR, *C*-glycosylflavones, rotamers, swertisin, embinoidin, *Anthurium aripoense*

## Abstract

Two known *C*-glycosylflavones, swertisin and embinoidin, were isolated from the leaves of *Anthurium aripoense*, and characterized by room temperature 1D and 2D NMR experiments. At this temperature, the ^1^H- and ^13^C-NMR spectra of these *C*-glycosylflavones revealed doubling of signals, which suggested the presence of two rotamers in solution. Variable-temperature (VT) ^1^H-NMR studies supported this hypothesis. The T-ROESY data, in addition to the theoretical (MM2) calculations utilizing the Chem3D Pro software, confirmed the hypothesis that the two rotamers interchange via rotation about the *C*-glycosidic bond.

## 1. Introduction

*Anthurium* is a genus of evergreen, climbing or epiphytic herbs which belongs to the Araceae [1], and has reported folkloric uses, which include hallucinogens, insecticides, oral contraceptives, rheumatoid arthritis treatments and skin care agents [2,3,4]. *Anthurium aripoense* is a terrestrial or short climbing herb found in Trinidad and Venezuela [5,6]. A survey of the literature revealed that there has been no phytochemical investigation of this species. Thus, the chemical constituents of this plant were investigated.

Two known *C*-glycosylflavones, swertisin and embinoidin [7], were isolated from the leaves of *Anthurium aripoense*. Both compounds showed duplication of signals in the ^1^H- and ^13^C-NMR spectra at room temperature. Similar observations had been made earlier on another *C*-glycosylflavone, spinosin, and attributed to the presence of two rotamers which were slowly interconverting via rotation about the *C*-glycosidic bond [8]. 

Rotational isomerism describes the phenomenon of rotation about a single bond in a molecule. Rotamers result when rotation is hindered by a rotational energy barrier [9]. Some interactions that influence the stability of rotamers include double bond character due to resonance, intramolecular hydrogen bonding and steric repulsions between adjacent atoms [10]. For the purpose of this study, steric hindrance proved to be the dominating effect. 

In this investigation, the structure of the two compounds was confirmed with the aid of 2D NMR experiments while the origin of signal doubling was investigated using variable-temperature (VT) ^1^H-NMR studies in combination with theoretical calculations. 

## 2. Results and Discussion

The structures of swertisin (**1**) and embinoidin (**2**) were confirmed and spectra assigned by a combination of ^1^H-^1^H COSY [11], HSQC [12] and HMBC [13] spectra. T-ROESY spectra [14] were obtained to aid in interpretation of the origins of the spectral doubling. Complete spectral assignments for the flavone moieties of swertisin (**1**) and embinoidin (**2**) are given in Table 1, while the spectral data for the sugar moieties are given in Table 2. 

**Figure 1:**
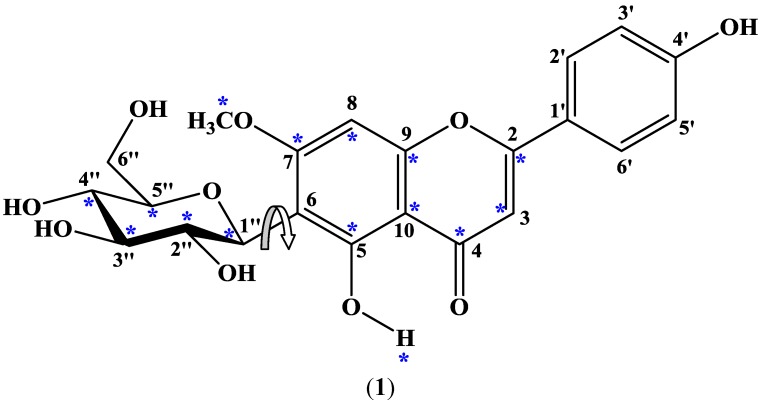
Diagram of swertisin showing atoms with duplicated signals indicated by asterisks; the arrow indicates rotation of the *C*-glycosidic bond.

**Figure 2:**
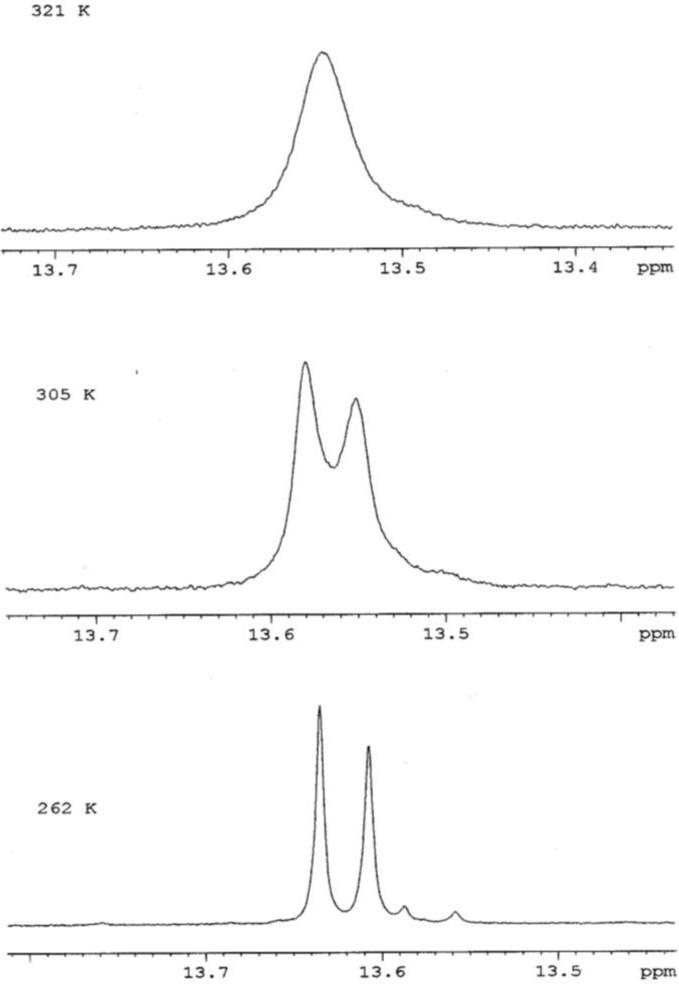
Effect of Temperature on the 5-OH signal markers in the ^1^H-NMR spectrum of swertisin (1).

The structure of swertisin (**1**) is shown in Figure 1, with carbons and the 5-OH proton giving rise to doubled signals marked with asterisks. The ^1^H-NMR spectrum of swertisin (**1**) in DMSO-*d*_6_ at 289 K showed that the relative proportion of the major and minor rotamers was 1.00:0.82. The duplication of the signals of the sugar unit and of carbons close to the *C*-glycosidic bond implied that there was an energy barrier about this bond that prevented fast exchange between the two rotamers. VT ^1^H-NMR studies were conducted on swertisin (**1**) to confirm this supposition. The VT ^1^H-NMR studies on swertisin (**1**) were not performed using DMSO-*d*_6_ as the solvent because the 5-OH signal markers were not detected individually just above the freezing temperature of DMSO-*d*_6_. On lowering the temperature below 292 K, the sample froze. Therefore, swertisin (**1**) was dissolved in (CD_3_)_2_CO-*d*_6_/DMSO-*d*_6_ (1:1) which had a freezing temperature of 258 K, thereby allowing the 5-OH signal markers to be individually detected. At 262 K and using the 5-OH signals as markers, the two rotamers of swertisin (1), with a relative proportion of 1.00:0.82, were independently detected since they were in slow exchange. At 305 K, the signals for the two rotamers moved even closer but were still detected. Eventually, the two signals coalesced to a single peak at ca. 13.54 ppm at a Coalescence Temperature, *T*_c_, of 321 K (Figure 2). The coalescence of signals was observed for the entire ^1^H-NMR spectrum, although the other pairs of signals coalesced at lower temperatures, reflecting their smaller chemical shift differences.

**Table 1:** ^1^H-NMR (600 MHz) and ^13^C-NMR (150 MHz) spectral data of the flavone nucleus of swertisin (**1**) and embinoidin (**2**) in DMSO-*d*_6_ (δ in ppm, *J* in Hz).

Position	1		2	
	δ_C_	δ_H_	δ_C_	δ_H_
C-2	164.01	―	163.39	―
	163.87	―	163.25	―
C-3	102.79	6.85 ( *s* )	103.78	6.95 ( *s* )
		6.83 ( *s* )	103.70	6.93 ( *s* )
C-4	182.21	―	182.02	―
	181.88	―	182.34	―
C-5	159.57	―	159.66	―
	160.31	―	160.53	―
C-6	109.65	―	108.62	―
			108.67	―
C-7	164.92	―	165.14	―
	163.71	―	163.86	―
C-8	91.00	6.82 ( *s* )	90.83	6.81 ( *s* )
	90.16	6.83 ( *s* )	90.37	6.84 ( *s* )
C-9	156.80	―	157.12	―
	156.70	―	157.01	―
C-10	104.07	―	104.24	―
	104.58	―	104.49	―
C-1′	120.37	―	122.69	―
C-2′, C-6′	128.52	7.96 ( *d*, 8.9)	128.37	8.08 ( *d*, 8.5)
C-3′, C-5′	116.16	6.92 ( *d*, 8.9)	114.61	7.13 ( *d*, 8.5)
C-4′	162.12	―	162.41	―
7-OMe	56.24	3.87 ( *s* )	56.13	3.92 ( *s* )
	56.48	3.96 ( *s* )	56.55	3.91 ( *s* )
4′-OMe	―	―	55.60	3.87 ( *s*)
5-OH	―	13.52 ( *s* )	―	13.46 ( *s* )
	―	13.54 ( *s* )	―	13.58 ( *s* )

* The small δ_C_ and δ_H_ made it difficult to associate a specific set of signals from the flavone nucleus with one of the sets of signals from the saccharide moiety.

**Table 2:** ^1^H-NMR (600 MHz) and ^13^C-NMR (150 MHz) spectral data of the sugar moieties of swertisin (**1**) and embinoidin (**2**) in DMSO-*d*_6_ (δ in ppm, *J* in Hz).

Position	1		2	
	δ_C_	δ_H_	δ_C_	δ_H_
**6-*C*-Glc**				
C-1″	72.82 ^a^	4.58 ( *d* , 10)	71.02 ^a^	4.68 ( *d* , 8.8)
	72.58 ^b^	4.60 ( *d* , 10)	70.70 ^b^	4.70 ( *d* , 8.8)
C-2″	69.62 ^a^	4.19 ( *dt* , 10, 2.6)	80.73 ^a^	4.47 ( *t* , 8.8)
	70.27 ^b^	4.00 ( *dt* , 10, 2.6)	81.21 ^b^	4.30 ( *t* , 8.8)
C-3″	79.08 ^a^	3.18 ( *m* )	78.29 ^a^	3.42 ( *m* )
	78.38 ^b^	3.18 ( *m* )	78.69 ^b^	3.44 ( *m* )
C-4″	70.92 ^a^	3.09 ( *m* )	70.45 ^a,b^	3.15 ( *m*)
	70.83 ^b^	3.09 ( *m* )		
C-5″	81.68 ^a^	3.16 ( *m* )	81.60 ^a^	3.16 ( *m* )
	81.86 ^b^	3.16 ( *m* )	81.93 ^b^	3.18 ( *m* )
C-6″	61.75 ^a,b^	3.70 ( *dd* , 12.9, 4.3),	61.45 ^a,b^	3.69 ( *dd* , 13, 4.4),
		3.37 ( *m* )		3.38 ( *m* )
**2″-*O*-Glc**				
C-1‴	―	―	105.42 ^a^	4.16 ( *d* , 8.8)
			105.25 ^b^	4.18 ( *d* , 8.8)
C-2‴	―	―	74.54 ^a^	2.85 ( *dt* , 8.8, 2.6)
			74.70 ^b^	2.85 ( *dt* , 8.8, 2.6)
C-3‴	―	―	76.32 ^a^	3.05 ( *m* )
			76.37 ^b^	3.06 ( *m* )
C-4‴	―	―	69.16 ^a^	3.02 ( *m* )
			69.46 ^b^	2.96 ( *m* )
C-5‴	―	―	76.42 ^a^	2.57 ( *dt* , 8.8, 2.6)
			76.68 ^b^	2.77 ( *dt* , 8.8, 2.6)
C-6‴	―	―	60.07 ^a^	3.16 ( *m*), 2.96 (*m* )
			60.60 ^b^	3.20 ( *m* )

^a^ ^13^C- and ^1^H-NMR saccharide signals for one rotamer; ^b^^13^C- and ^1^H-NMR saccharide signals for the second rotamer.

The free energy of activation for the interconversion between the two unequally populated rotamers of swertisin (**1**) can be calculated using Eyring’s equations (**a** and **b**) as modified by Shanan-Atidi and Bar-Eli [15]:


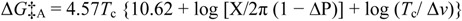
(**a**)


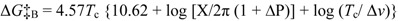
(**b**)

where X = 2πτ∆*v* and ∆P = P_A_ − P_B_.

P_A_ and P_B_ represent the population of the conformers A and B (P_A_ > P_B_, P_A_ + P_B_ = 1), respectively, and τ is the mean lifetime. *T*_c_ and ∆*v* are the coalescence temperature and the chemical shift difference between conformers A and B, respectively. X is obtained using equation (**c**):


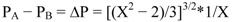
(**c**)

From the ^1^H-NMR spectrum at 262 K, the frequency difference, Δ*ν*, between the 5-OH signals was 11.13 Hz (11.13 s^−1^). The Coalescence Temperature, *T*_c_, was 321 K.


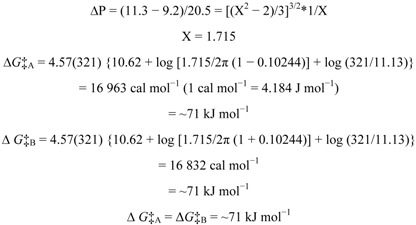


The VT ^1^H-NMR studies confirmed the hypothesis that the doubling of signals in the ^1^H- and ^13^C-NMR spectra at 262 K was due to the presence of two rotamers of swertisin (**1**) separated by a relatively high energy barrier.

The data derived from the T-ROESY spectrum suggested that the two rotamers slowly rotated about the *C*-glycosidic (C-1″—C-6) bond. Weak cross-peaks were observed between the respective methoxyl protons (7-OMe), and the H-1″^a^ and H-2″^b^ protons. This observation indicated that *β*-D-glucose rotates about the C-1″—C-6 bond. Therefore, in one rotamer, the H-1″ proton is oriented *syn* to the 7-OMe group whereas, in the other rotamer, the H-2″ proton is oriented *syn* to the 7-OMe group. 

**Figure 3:**
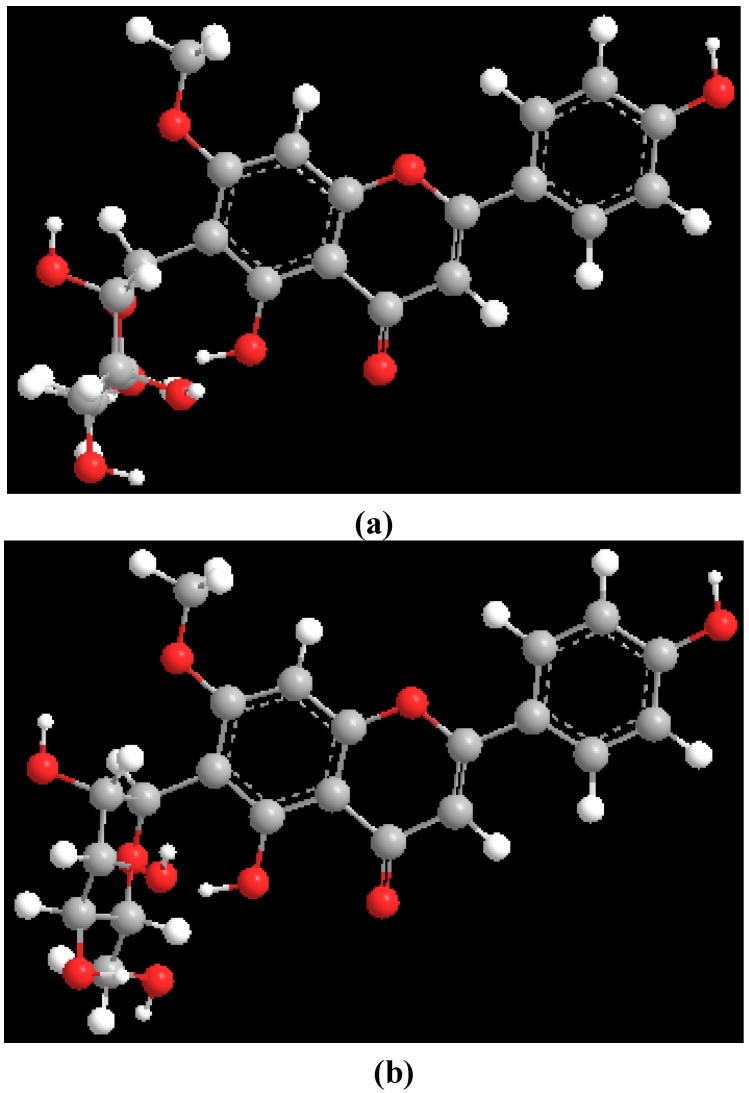
Molecular models of the rotamers of swertisin.

Theoretical (MM2) calculations of swertisin (**1**) were performed utilizing the ChemDraw 3D Pro software, in which the dihedral angle was defined by highlighting four contiguous atoms [H-1", C-1", C-6 and C-7 (Figure 1)]. Two minimum energy conformations (Figure 3) were separated by a rotational energy barrier (Δ*G*^#^_rot_) of 70 kJ mol^−1^. The experimental Δ*G*^#^_rot_ (~71 kJ mol^−1^) and theoretical Δ*G*^#^_rot_ (70 kJ mol^−1^) of swertisin (**1**) were essentially equal. Therefore, the theoretical (MM2) calculations in conjunction with the VT ^1^H-NMR studies and T-ROESY data of swertisin (**1**) supported the proposal that the two rotamers of swertisin (**1**) interchange by rotation about the *C*-glycosidic (C-1″—C-6) bond.

**Figure 4:**
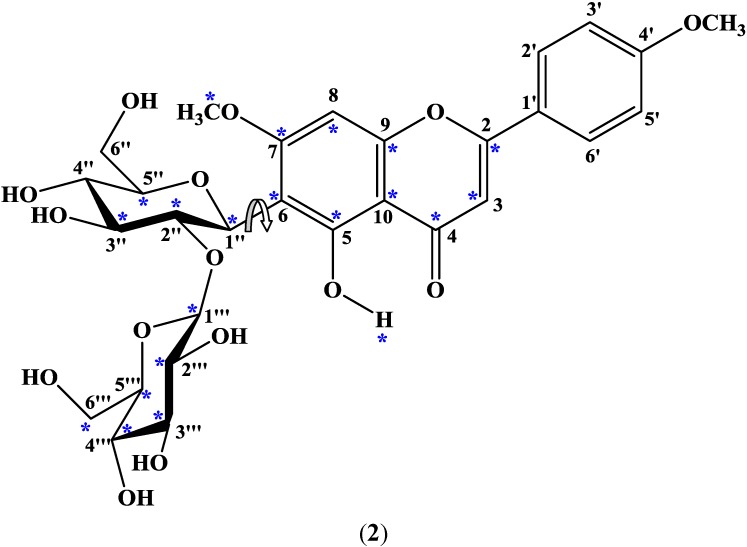
Diagram of embinoidin showing atoms with duplicated signals indicated by asterisks; the arrow indicates rotation of the *C*-glycosidic bond.

The structure of embinoidin (**2**) is shown in Figure 4, with carbons and the 5-OH proton giving rise to doubled signals marked with asterisks. At 305 K, the ^1^H-NMR spectrum of embinoidin (**2**) in DMSO-*d*_6_ indicated a doubling of the signals which was attributed to the presence of two rotamers, with a relative proportion of 1.00:0.97, in solution. The duplicated ^1^H-NMR signals suggested that, at 305 K, there were two rotamers separated by an energy barrier about the *C*-glycosidic bond which hindered rotation between the rotamers. VT ^1^H-NMR studies were performed on embinoidin (**2**) in order to confirm this hypothesis.

**Figure 5:**
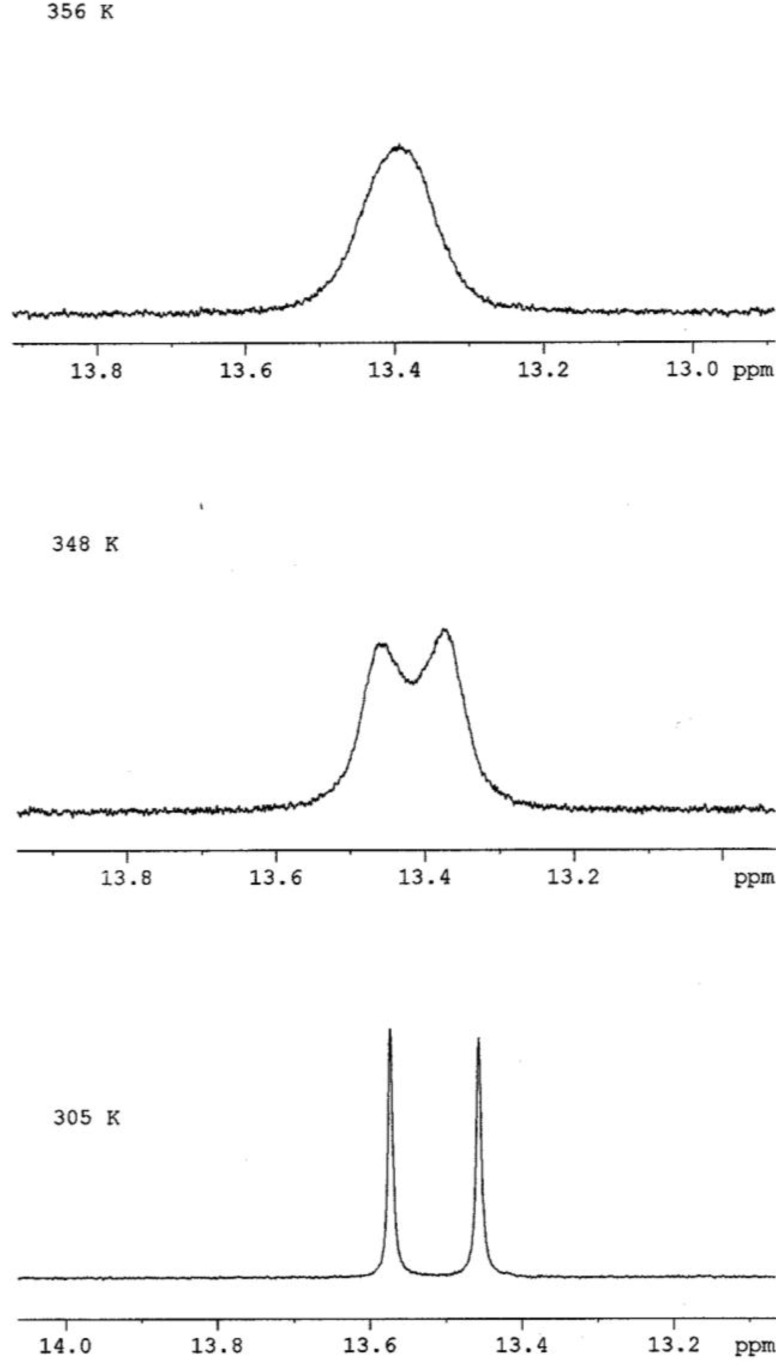
Effect of Temperature on the 5-OH signal markers in the ^1^H-NMR spectrum of embinoidin (**2**).

At 305 K and using the 5-OH signals as markers, the two rotamers of embinoidin (**2**) in DMSO-*d*_6_ were in slow exchange so that each rotamer was detected independently. At 348 K, the signals for the two rotamers were still individually detected, although, the two 5-OH signals had broadened and moved closer together. Eventually, the two signals coalesced to a single peak at ca. 13.4 ppm at a Coalescence Temperature, *T*_c_, of 356 K (Figure 5). The coalescence of signals was observed for the entire ^1^H-NMR spectrum, although the other pairs of signals coalesced at lower temperatures, reflecting their smaller chemical shift differences. The free energy of activation for the interconversion between the two rotamers of embinoidin (**2**) was also calculated using Eyring’s equations (**a** and**b**) as modified by Shanan-Atidi and Bar-Eli [15].

From the ^1^H-NMR spectrum at 305 K, the frequency difference, Δ*ν*, between the 5-OH signals was 46.6 Hz (46.6 s^−1^). The Coalescence Temperature, *T*_c_, was 356 K.


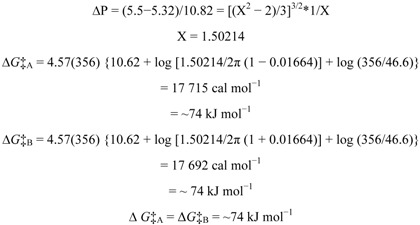


Since the relative proportion of the two rotamers was approximately 1:1 at 305 K, the free energy of activation for rotation, Δ*G*‡_rot_, was also calculated using Eyring equation for equally populated rotamers [16]. H. S. Gutowsky showed that the rate of rotation, *k*_c_, at the temperature of coalescence, *T*_c_, 356 K, can be calculated using the following equation [16]:


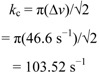


The free energy of activation for rotation, Δ*G*‡_rot_, at 356 K was calculated using the Eyring equation [16]: 


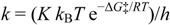


where *k* is the rate constant, *k*_B_ is Boltzmann’s constant, *h* is Planck’s constant, *K* is the transmission coefficient, *T* is the temperature in K, *R* is the universal gas constant and Δ*G*‡ is the free energy of activation.

Assuming the transmission coefficient, *K*, to be unity, converting natural log (ln) to log_10_, and substituting *k*_c_ and *T*_c_ into the Eyring equation, this equation becomes [16]: 


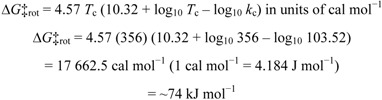


The VT ^1^H-NMR studies confirmed the hypothesis that the doubling of signals in the ^1^H- and ^13^C-NMR spectra at 305 K was due to the presence of two rotamers of embinoidin (**2**) separated by a relatively high energy barrier.

The data derived from the T-ROESY spectrum again suggested that the two rotamers slowly rotated about the *C*-glycosidic bond. Weak cross-peaks were observed between the respective methoxyl protons (7-OMe), and both signals of H-1″^a^ and H-2″^b^. This observation indicated that *β*-D-glucose, which is directly attached to the flavone nucleus, rotates about the *C*-glycosidic bond. Therefore, in one rotamer, the H-1″ proton is oriented *syn* to the 7-OMe group whereas, in the other rotamer, the H-2″ proton is oriented *syn* to the 7-OMe group. Stronger cross-peaks were observed between the H-2″^a^ and H-1‴^a^ signals, and between the H-2″^b^ and H-1‴^b^ proton signals. Thus, in each rotamer, the rotation about the C-2″―O―C-1‴ bond brings the H-1‴ proton in close proximity to the H-2″ proton.

**Figure 6:**
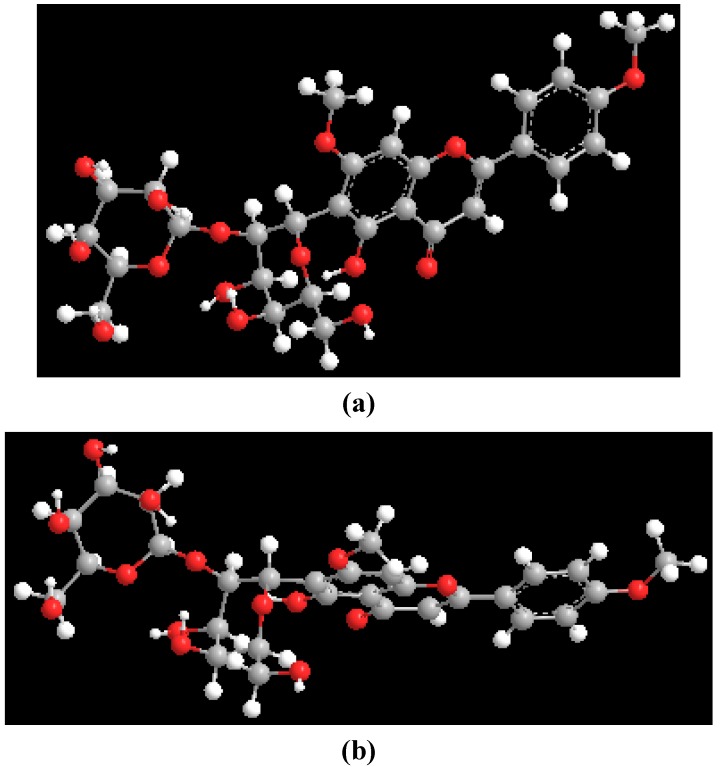
Molecular models of the rotamers of embinoidin.

Theoretical (MM2) calculations of embinoidin (**2**) were conducted utilizing the ChemDraw 3D Pro software, where the four contiguous atoms of H-1", C-1", C-6 and C-7 defined the dihedral angle. Two minimum energy conformations (Figure 6) were separated by Δ*G*^#^_rot_ of 69 kJ mol^−1^. The experimental Δ*G*^#^_rot_ (~74 kJ mol^−1^) and theoretical Δ*G*^#^_rot_ (69 kJ mol^−1^) of embinoidin (**2**) were close in value. Therefore, the theoretical (MM2) calculations in conjunction with the VT ^1^H-NMR studies and T-ROESY data of embinoidin (**2**) supported the proposal that the two rotamers of embinoidin (**2**) interchange by rotation about the *C*-glycosidic bond.

## 3. Experimental

### 3.1. General

Mass spectral data was obtained using a Bruker Daltonics micrOTOF-Q ESI mass spectrometer, and the UV-visible spectra were recorded on a Varian CARY 50 Conc UV-visible spectrophotometer. IR spectra were acquired on Perkin Elmer FTIR RX1 spectrophotometer. Column chromatography was carried out on Merck silica gel 60 (70–230 mesh). At room temperature, the ^1^H-, ^13^C-, ^1^H-^1^H COSY, HSQC, HMBC and T-ROESY NMR experiments were performed using a Bruker Avance DRX-600 spectrophotometer which was equipped with a 5 mm PATXI indirect detection probe with a Z gradient coil (^1^H 90° pulse width = 9.90 μs, ^13^C 90° pulse width = 12.55 μs). The VT ^1^H-NMR data were acquired on a Bruker-Avance DRX-400 spectrophotometer equipped with a 5 mm ^1^H/^13^C/^19^F/^31^P probe and a BVT-3300 VT controller for temperature regulation and measurement. The samples were dissolved in DMSO-*d*_6_ or (CD_3_)_2_CO-*d*_6_/DMSO-*d*_6_ (1:1), chemical shifts are reported in parts per million (ppm) downfield from tetramethylsilane (0 ppm) as the internal standard, and coupling constants (*J*) are recorded in Hertz (Hz). Operating parameters for the ^1^H-detected experiments were an F2 (^1^H) spectral width of 12335.526 Hz with 64 K data points, whereas the parameters for the ^13^C-detected experiments were an F1 (^13^C) spectral width of 36057.691 Hz with 64 K data points, a 30° pulse width and 8K scans. For the high resolution HSQC spectra, F1 was 28673.971 Hz and F2 was 7936.508 Hz, 256 time increments were linear predicted to 1024, with 16 transients per increment, and for HMBC spectra, F1 was 34711.465 Hz and F2 was 7936.708 Hz with 32 transients per increment and 256 time increments linear predicted to 1024. The ^1^H-^1^H COSY spectra were processed in the absolute value mode. Phase-sensitive T-ROESY spectra used the same ^1^H spectral windows and F2 data points with 256 increments linearly predicted to 1024 with a mixing time of 0.2 s and a relaxation delay of 2.0 s.

### 3.2. Plant Material, Extraction and Isolation

The leaves of *A. aripoense*, growing at an altitude of ca. 800 m, were collected at El Cerro del Aripo, Trinidad, on January 2008. The plant was identified by Mr. Winston Johnson of the National Herbarium of Trinidad and Tobago, where a voucher specimen (TRIN 36517) was deposited.

The dried leaves of *A. aripoense* (2.00 kg) were extracted exhaustively with 100% MeOH (20 L, 10 a.m., 28 °C) to yield after solvent removal the methanolic crude extract (179.61 g). This extract was suspended in MeOH/H_2_O (90:10, 500 mL) and partitioned sequentially with petroleum ether, EtOAc and *n*-BuOH (700 mL × 5, 700 mL × 5, 700 mL × 2, respectively) to yield after concentration the petroleum ether (57.45 g), EtOAc (18.15 g) and *n*-BuOH (22.24 g) soluble fractions.

A portion of the EtOAc fraction (18.00 g) was subjected to isocratic column chromatography on silica gel eluting with petroleum ether/EtOAc (3:1) to yield five fractions. The fifth fraction (14.99 g) was subjected to repetitive silica gel column chromatography using CHCl_3_/MeOH mixtures of (96:4), (85:15) and (80:20), respectively, to eventually yield four fractions. The second fraction (3.40 g) was further separated isocratically on a silica gel column using EtOAc/MeOH (90:10) to afford a fraction (0.07 g) which when concentrated under reduced pressure yielded swertisin (**1**, 30.9 mg).

A portion of the *n*-BuOH fraction (22.00 g) was subjected to isocratic column chromatography on silica gel using CHCl_3_/MeOH (75:25) to yield five fractions. The fifth fraction (4.18 g) was further fractionated on a silica gel column using CHCl_3_/MeOH (75:25) to afford four fractions. When concentrated under reduced pressure, the third fraction yielded embinoidin (**2**, 45.4 mg).

### 3.3. Spectral Data

*Swertisin* (**1**). Bright yellow amorphous powder; UV λ_max_ (DMSO) nm (log ε): 340 (5.79); IR (nujol) ν_max_ cm^−1^: 3363, 1652, 1603; ^1^H- and ^13^C-NMR (DMSO-*d*_6_): see Table 1 and Table 2; HRESIMS *m/z* 447.1249 [M + H]^+^ (calc. for C_22_H_22_O_10_, 447.1286).

*Embinoidin* (**2**). Pale yellow amorphous powder; UV λ_max_ (DMSO) nm (log ε): 325 (3.64); IR (nujol) ν_max_ cm^−1^: 3365, 1653, 1603; ^1^H- and ^13^C-NMR (DMSO-*d*_6_): see Table 1 and Table 2; HRESIMS *m/z* 623.1872 [M + H]^+^ (calc. for C_29_H_34_O_15_, 623.1970).

## 4. Conclusions

Two *C*-glycosylflavones were isolated from the leaves of *A. aripoense* and identified as swertisin (**1**) and embinoidin (**2**). The ^1^H-NMR spectra of the *C*-glycosylflavones revealed a duplication of signals which suggested the presence of two rotamers in solution. VT ^1^H-NMR studies confirmed that there were two rotamers of swertisin (**1**) and embinoidin (**2**) which interchanged via rotation about the *C*-glycosidic bond. 
